# Migraine associated with early onset postpartum depression in women with major depressive disorder

**DOI:** 10.1007/s00737-021-01131-6

**Published:** 2021-04-21

**Authors:** Katherine Gordon-Smith, Paul Ridley, Amy Perry, Nicholas Craddock, Ian Jones, Lisa Jones

**Affiliations:** 1 Psychological Medicine, University of Worcester, Henwick Grove, Worcester, WR2 6AJ UK; 2 GP Speciality School, Health Education North West, North West, UK; 3 Division of Psychiatry and Clinical Neurosciences, School of Medicine, Cardiff University, Cardiff, UK

**Keywords:** Major depressive disorder, Migraine, Women, Lifetime reproductive events, Postpartum depression

## Abstract

Major depressive disorder (MDD) and migraine are both more common among women than men. Women’s reproductive years are associated with increased susceptibility to recurrence of both conditions, suggesting a potential role of sex hormones in aetiology. We examined associations between comorbid migraine and clinical features of MDD in women, including relationships with lifetime reproductive events such as childbirth. Lifetime clinical characteristics and reproductive events in a well-characterised sample of 222 UK women with recurrent MDD, with (*n* = 98) and without (*n* = 124) migraine were compared. Women had all been recruited as part of a UK-based ongoing programme of research into the genetic and non-genetic determinants of mood disorders. Multivariate analysis showed a specific association between the lifetime presence of migraine and postpartum depression (PPD) within 6 weeks of delivery (OR = 2.555; 95% CI: 1.037–6.295, *p* = 0.041). This association did not extend to a broader definition of PPD with onset up to 6 months postpartum. All other factors included in the analysis were not significantly associated with the presence of migraine: family history of depression, younger age at depression onset, history of suicide attempt and severe premenstrual syndrome symptoms. The finding that women with MDD and comorbid migraine may be particularly sensitive to hormonal changes early in the postpartum period leads to aetiological hypotheses and suggests this group may be useful for future studies attempting to characterise PPD and MDD phenotypes. The refinement of such phenotypes has implications for individualising risk and treatment and for future biological and genetic studies.

## Introduction

The lifetime prevalence rate of major depressive disorder (MDD) is estimated to be twice as high in women compared to men (Kessler et al. 1993), with epidemiological studies showing increased risk of depressive episodes and symptoms in the reproductive phases of women’s lives, including the early postpartum period (O’Hara and Swain 1996; Munk-Olsen et al. 2006) and during the menopausal transition (Freeman et al. 2006). MDD is highly comorbid with migraine (Breslau et al. 2003; Nguyen and Low 2013), and similarly, there is extensive literature demonstrating migraine is more prevalent among women compared to men (Vetvik and MacGregor 2017). The female to male ratio of migraine prevalence is not consistent across all age ranges and peaks during the reproductive years (Victor et al. 2010; Buse et al. 2013). During women’s reproductive years, peaks in susceptibility to migraine occur during times of major hormonal changes. The menstrual cycle is linked with the onset of migraine and women are more susceptible to migraine attacks in the 4 day window surrounding the onset of menses (Granella et al. 2000; Vetvik et al. 2014). There is a reported improvement of migraine symptoms during pregnancy depending on the type of migraine (MacGregor 2007) followed by an increase in the postpartum period (Kvisvik et al. 2011).

MDD and migraine are both multifactorial conditions, shown to have a bidirectional relationship, in which each disorder increases relative risk for first onset of the other (Breslau et al. 2000). The accepted view is that overlapping underlying pathophysiology is likely to play a role in the association between the two conditions, for example, dysregulation of the serotonergic neurotransmitter system (Ressler and Nemeroff 2000; Deen et al. 2017). There is also evidence of shared genetic determinants (Yang et al. 2018). The disproportionate prevalence of both conditions in women suggests sex hormones plausibly have a contributing role.

Migraine comorbidity in MDD has previously been found to be associated with more frequent and longer depressive episodes, and a higher severity of depressive symptoms (Oedegaard and Fasmer 2005; Hung et al. 2015). The findings of a recent population-based study in Switzerland also suggest differential associations between the subtypes of MDD and the presence of migraine. Specifically melancholic but not atypical MDD was found to be associated with the lifetime presence of migraine (Pisanu et al. 2020). However, there is a paucity of research examining the association between comorbid migraine and MDD in women, and specifically relationships with lifetime reproductive events. Such research has the potential to increase understanding of the gender-specific associations between migraine and MDD and shine a light on the aetiology of these conditions.

The aim of the current study was to investigate the lifetime relationships between migraine and clinical features of MDD, including lifetime reproductive events, in a well-characterised UK sample of women.

## Methods

Data were collected as part of an ongoing programme of research into the genetic and non-genetic determinants of mood disorders including MDD. The research programme has UK National Health Service (NHS) Health Research Authority (HRA) approval–Research Ethics Committee (REC) reference (MREC/97/7/01) and local approvals in all participating NHS Trusts/Health Boards.

### Recruitment of participants

Participants were recruited systematically via UK NHS psychiatric services or non-systematically through the research team website, advertisements in local and national media and through patient support organisations.

The research programme inclusion criteria required participants to be aged 18 years or over, able to provide written informed consent, meet DSM-IV criteria for major affective disorder and for mood symptoms to have started before the age of 65 years. Individuals were excluded if they: (i) experienced affective illness only as a result of alcohol or substance dependence or (ii) experienced affective illness only secondarily to medical illness or medication. All participants were of UK White ethnicity due to a key focus of the research programme on genetic causes of affective disorders. Additional exclusion criteria for participants with MDD recruited into the research programme were (i) a first- or second-degree relative with a diagnosis of bipolar affective disorder, schizophrenia, schizotypal disorder, delusional disorder, acute and transient psychotic disorders or schizoaffective disorder, and (ii) a history of experiencing mood incongruent psychosis or psychosis outside of mood episodes.

Participants included in the current study were unrelated women with a DSM-IV lifetime diagnosis of recurrent MDD, who were assessed for the presence of migraine (*n* = 288).

### Psychiatric assessment

Participants were interviewed using the schedules for clinical assessment in neuropsychiatry (SCAN) (Wing et al. 1990), which provides detailed information about lifetime psychopathology. Where available, information was also gathered from review of psychiatric and general practice case-notes and combined with the interview data in order to establish a best-estimate lifetime diagnosis according to DSM-IV criteria. These data were also used to rate key lifetime demographic and clinical variables, for example, age of depression onset, number of depressive episodes, history of psychiatric admissions, family history of depression (defined as at least one first- or second-degree relative with a history of depression) and lifetime suicidal behaviour. Level of function during each participant’s lifetime worst episode of depression was assessed using the Global Assessment Scale (GAS) (Endicott et al. 1976). The GAS measures overall functioning during a specified time frame on a continuum from illness to health with scores ranging from 1 to 100 (lowest-highest level of functioning). In cases where there was doubt, diagnostic and clinical ratings were made by at least two members of the research team blind to each other’s rating, and consensus was reached via discussion where necessary. Inter-rater reliability was formally assessed using 20 random cases. Mean kappa statistics were 0.85 for DSM-IV diagnosis and ranged between 0.81 and 0.99 for other key clinical categorical variables. Mean intra-class correlation coefficients were between 0.91 and 0.97 for key clinical continuous variables. Team members involved in the interview, rating and diagnostic procedures were all research psychologists or psychiatrists.

### Lifetime reproductive events

At interview, parous women (*n* = 161) were asked about the lifetime occurrence of mood episodes in each perinatal period (during pregnancy up to 6 months postpartum). A rating was made of the lifetime most impairing episode of perinatal depression categorised as one of: (i) no perinatal depressive episodes; (ii) depressive episode with onset during pregnancy; (iii) postpartum depression (PPD) with onset up to 6 months following delivery. A narrow onset criterion for PPD restricted to the first 6 weeks following delivery was also rated to be consistent with both DSM-5 and ICD-11 definitions of the postpartum period.

As part of a wider set of questionnaires completed and returned after the interview (response rate 85%), women were asked a series of questions about age at menarche, menstrual cycle regularity and premenstrual symptoms. Four items were used to assess the psychological aspects of premenstrual syndrome (PMS): *Just before the start of your period, do/did you usually: (i) have less energy than usual or get tired more easily? (ii) feel more sad, blue or depressed? (iii) feel more irritable or get upset more easily? (iv) have any other changes in health or mood?* Four possible responses to these items were: ‘No’, ‘A Little’, ‘Some’ and ‘A lot’ (rated 0–3) which for the purpose of analyses were dichotomised into ‘No’ or ‘A Little’ (rated 0) vs. ‘Some’ or ‘A lot’ (rated 1). These four items were also summed to form a short premenstrual symptom scale with scores ranging from 0 to 12.

### Migraine assessment 

The lifetime presence of migraine was assessed using a self-report bespoke questionnaire, completed at the end of the interview and returned directly to the interviewer. The questionnaire consisted of 10 items relating to migraine frequency, character, duration, severity, associated symptoms and aggravating and relieving factors. Participant responses were used to make a lifetime diagnosis of migraine according to criteria adapted from the International Headache Society diagnostic criteria second edition (International Headache Society 2004). Women were stratified into those with a lifetime history of migraine (MDD + m) (*n* = 98, *n* = 69 parous) and those without a history of any headaches or migraine (MDD − m) (*n* = 124, *n* = 92 parous). Those with a history of tension headaches or headaches not reaching migraine criteria were excluded from the analysis (*n* = 66).

### Analysis

As the majority of data were not normally distributed, non-parametric statistical tests were used. For comparisons of demographic and clinical features between the MDD + m and MDD − m groups, chi-squared tests and Mann–Whitney *U* tests were used to compare categorical and continuous variables, respectively. To identify which lifetime clinical features best predicted the presence of migraine in MDD, all lifetime clinical variables significant at the 5% level (*p* < 0.05) were included as explanatory variables in a binary logistic regression model (enter method), with MDD + m vs. MDD − m as the outcome variable. All demographic variables found to be significant at *p* < 0.05 were also included as explanatory variables. We also included age at interview in the model as a potential confounder. Statistical analyses were carried out using the Statistical Package for the Social Sciences (SPSS) version 27.0.

## Results

### Demographic characteristics

There were no significant associations between the presence of migraine in the women with MDD and the demographic characteristics examined (Table 1).

**Table 1:** Demographic characteristics of women with MDD with and without migraine

	MDD + m (*n* = 98)	MDD − m (*n* = 124)	*p*-value
Age at interview, years, median (IQR)	48 (17) (*n* = 98)	52 (19) (*n* = 124)	0.084
Systematically recruited, % (*n*)	40.8 (40/98)	53.7 (66/123)	0.058
Highest occupational level (professional), % (*n*)	55.9 (52/93)	56.9 (66/116)	0.887
Highest educational level (higher education), % (*n*)	27.5 (25/91)	25.2 (28/111)	0.718
Marital history (has married), % (*n*)	78.6 (77/98)	85.5 (106/124)	0.179
Ever regular tobacco smoker, % (*n*)	54.8 (51/93)	43.3 (52/120)	0.096
Heaviest weekly alcohol consumption, units, median (IQR)	11 (30) (*n* = 88)	10 (18) (*n* = 121)	0.104

*MDD* + *m*, major depressive disorder with migraine; *MDD − m*, major depressive disorder without migraine; *IQR*, inter-quartile range. Numbers vary for each variable due to missing data

### Clinical features

Women with MDD + m had a significantly younger median age of depression onset, defined as the age at first impairment due to depression (23 vs. 27 years, *p* = 0.006), and were more likely to have a family history of depression (94.1% vs. 80.6%, *p* = 0.007) and to have attempted suicide (41.2% vs. 22.0%, *p* = 0.002) than women with MDD − m (Table 2). The two groups were however not significantly different in terms of lifetime number of depressive episodes per illness year, history of psychiatric admission, lifetime presence of psychotic symptoms and severity of functional impairment during the worst episode of depression as measured according to the GAS.

**Table 2:** Lifetime clinical characteristics and reproductive events of women with MDD with and without migraine

	MDD + m (*n* = 98)	MDD − m (*n* = 124)	*p*-value
Age of depression onset, years, median (IQR)	23 (10) (*n* = 98)	27 (15) (*n* = 123)	**0.006**
No. depressive episodes per illness year, median (IQR)	0.2 (0.2) (*n* = 97)	0.2 (0.3) (*n* = 123)	0.695
History of suicide attempt, % (*n*)	41.2 (40/97)	22.0 (27/123)	**0.002**
History of psychiatric admission, % (*n*)	43.5 (37/85)	43.6 (44/101)	0.996
History of psychosis, % (*n*)	12.2 (12/98)	13.1 (16/122)	0.847
Family history of depression^a^, % (*n*)	94.1 (80/85)	80.6 (79/98)	**0.007**
GAS lifetime worst in depressive episode, median (IQR)	35.5 (8) (*n* = 98)	35 (8) (*n* = 124)	0.470
Lifetime reproductive events			
Lifetime any perinatal depressive episode, % (*n*) (vs. no perinatal depressive episode)	58.0 (40/69)	54.3 (50/92)	0.647
Lifetime PPD within 6 months of delivery, % (*n*) (vs. no perinatal depressive episode or depressive episode in pregnancy)	50.7 (35/69)	42.4 (39/92)	0.294
Lifetime PPD within 6 weeks of delivery, % (*n*) (vs. no perinatal depressive episode, depressive episode in pregnancy or PPD later than 6 weeks postpartum)	44.9 (31/69)	27.2 (25/92)	**0.019**
First depressive episode within 6 months postpartum, % (*n*)	23.2 (16/69)	23.9 (22/92)	0.915
Age at menarche, years, median (IQR)	13 (3) (*n* = 89)	13 (2) (*n* = 101)	0.501
Lifetime regular menstrual cycle, % (*n*)	76.2 (64/84)	78.2 (79/101)	0.743
PMS have less energy than usual or get tired more easily—some or a lot, % (*n*) (vs. no or a little)	67.9 (55/81)	60.4 (58/96)	0.302
PMS feel more sad, blue or depressed—some or a lot, % (*n*) (vs. no or a little)	68.6 (59/86)	63.4 (59/93)	0.466
PMS feel more irritable or get upset more easily—some or a lot, % (*n*) (vs. no or a little)	81.2 (69/85)	68.7 (68/99)	0.053
PMS have any other changes in health or mood—some or a lot, % (*n*) (vs. no or a little)	63.0 (51/81)	46.7 (43/92)	**0.033**
Total PMS score (range 0–12), median (IQR)	9 (5) (*n* = 81)	8 (7) (*n* = 92)	**0.041**

*MDD* + *m*, major depressive disorder with migraine; *MDD − m*, major depressive disorder without migraine; *PPD*, postpartum depression; *IQR*, inter-quartile range; *GAS*, Global Assessment Scale; *PMS*, premenstrual syndrome. Significant results at *p* < 0.05 are shown in bold. Numbers vary for each variable due to missing data (perinatal episodes reported for parous women only)

^a^Family history of depression: defined as at least one first- or second-degree relative with depression

### Lifetime reproductive events

Parous women with MDD + m in the sample were significantly more likely to have experienced a lifetime episode of PPD within 6 weeks of delivery compared to parous women with MDD − m (44.9% vs. 27.2%, *p* = 0.019). This association did not extend to a broader definition of a lifetime episode of PPD within 6 months of delivery, or to an even broader definition of any lifetime perinatal depressive episode (defined as any lifetime depressive episode with onset either during pregnancy or up to 6 months postpartum). Women with and without migraine did not differ significantly in terms of the proportion who experienced their first episode of depression within 6 months postpartum (23.2% vs. 23.9%), age at menarche (13 years in both groups) or history of a regular menstrual cycle (76.2% vs. 78.2%, respectively). For one of the four PMS symptoms ‘other changes in health or mood’, a significantly higher proportion of women with MDD + m reported the symptom as occurring ‘some’ or ‘a lot’ compared to women with MDD − m (63.0% vs. 46.7%, *p* = 0.033). The other three PMS symptoms showed the same trend but were not significant. The median overall total PMS score was higher in the MDD + m group (9 vs. 8), just reaching statistical significance (*p* = 0.041).

The following demographic and lifetime clinical characteristics were included as explanatory variables in the multivariate analysis: age at depression onset, family history of depression, history of suicide attempt, lifetime PPD within 6 weeks of delivery and total PMS score. The single factor found to be significantly associated with the presence of migraine in women with MDD was PPD within 6 weeks of delivery (OR = 2.555; 95% CI: 1.037–6.295, *p* = 0.041). This explained 23.5% (Nagelkerke *R*^2^ = 0.235) of the variance.

## Discussion

This is the first study to date to investigate differences in a range of both clinical characteristics and lifetime reproductive events between women who have MDD with and without comorbid migraine. The presence of comorbid migraine was significantly associated with lifetime history of PPD within 6 weeks of delivery, even after controlling for other significant demographic and clinical differences between the groups in multivariate analysis.

Our findings suggesting an association between migraine and PPD overlap with those of a recent large Swedish population-based study to identify characteristics and predisposing factors associated with different perinatal depression trajectories (pregnancy depression, early postpartum onset, late postpartum onset and chronic depression) (Wikman et al. 2020). Migraine was one of the characteristics associated with all perinatal depression trajectories when compared with women defined as healthy (no depressive symptoms during pregnancy or postpartum and no history of depression). The results differ in that our findings suggest a specific association between migraine and depression with an early postpartum onset (within 6 weeks after delivery). The more broadly defined occurrences of PPD within 6 months of delivery and any perinatal depressive episode were not significantly associated with the presence of migraine in our sample. However, there are a number of methodological differences between the studies, including that our study focused specifically on women with MDD.

A small number of previous cohort studies, also not specifically examining women with MDD, have shown women with migraine to be at high risk for perinatal mental illness. Cripe et al. (2010) examined the relationship between history of migraine and depressive symptoms in pregnancy in a large cohort of Peruvian women (*n* = 2293) who were all interviewed within 4 days of delivery during their postpartum stay in hospital. The adjusted odds of moderate to severe depressive symptoms during pregnancy among women with a history of migraine (55.1%) were 2.06 times that of women without a history of migraine (36.7%). Similarly among a cohort of 1321 women interviewed during the first trimester of pregnancy in the United States, those with a history of migraine had an increased odds of moderate to severe depressive symptoms during pregnancy compared to those without a history of migraine (16.4% vs. 12.7%; OR: 1.60) (Orta et al. 2015). A prospective cohort study of 1423 pregnant women examining a wide range of potential risk factors for PPD including pre-pregnancy medical illness found women with PPD in comparison with women without PPD reported higher rates of pre-pregnancy migraines in univariate, but not multivariate, analyses (Katon et al. 2014).

Our finding of an association between history of migraine and PPD among women with MDD is likely to be due to a complex interaction of psychosocial and biological factors. Psychosocial factors might include, for example, the difficulties of living with migraine symptoms in the postpartum period increasing risk of depression. Potential neurobiological factors include overlapping mechanisms implicated in the pathophysiology of both migraine and PPD, including the hypothalamic–pituitary–adrenal (HPA) axis (Peres et al. 2001; Glynn et al. 2013), immune system (Bruno et al. 2007; Anderson and Maes 2013), neurotransmitter circuits (Hamel 2007; Moses-Kolko et al. 2008) and most obviously sex hormones. As highlighted in a recent review, fluctuations in hormone concentrations, in particular oestrogen, are thought to account for the higher prevalence ratio of migraine in women during the reproductive years (Vetvik and MacGregor 2017). Specifically, women with migraine have been found to have a faster rate of oestrogen decline in the late luteal phase of the menstrual cycle compared to healthy controls (Pavlović et al. 2016). Similarly, a hallmark of the postpartum period is the drop in oestrogen immediately after childbirth. Early work in this area found evidence that women with a history of PPD may be differentially sensitive to these rapid hormonal changes by demonstrating that the add-back and subsequent withdrawal of reproductive hormones increased depression scores only in women with a prior history of PPD (Bloch et al. 2000). Depressive symptoms peaked in intensity during the withdrawal phase in this sample. More recently, a genome-wide association study found that women with PPD had an increased sensitivity to oestrogen signalling, with significant gene expression changes in oestrogen-responsive genes (Mehta et al. 2014). Oestrogen-mediated epigenetic changes have also been found to be associated with PPD (Guintivano et al. 2014). In light of these findings, the existence of a hormone-sensitive PPD phenotype has been proposed (Schiller et al. 2015).

The association between history of migraine and PPD in our sample did not appear to be explained by women in the migraine group experiencing more severe or more frequent mood episodes over the course of their lives. A number of lifetime clinical characteristics, including number of episodes per illness year and level of functioning during the worst episode of depression, did not differ significantly between the migraine and no migraine groups. Furthermore, the association remained significant in multivariate analysis which controlled for earlier age of onset of depression and history of suicide attempts, both of which were significantly associated with the presence of migraine in univariate analysis. Our findings suggest this group of women with MDD and comorbid migraine may be particularly sensitive to hormonal changes early in the postpartum. Despite there being no difference in the age at menarche or proportion of women reporting regular menstrual cycles between the groups, women with a history of migraine did have a significantly higher overall PMS score but this did not remain significant in multivariate analysis. In our univariate analyses, women in the migraine group were also significantly more likely to report other changes in health or mood premenstrually, although we were unable to elicit whether or not this included migraine. For this reason and multicollinearity with total PMS score, we did not include this variable in the multivariate analysis.

Findings from the international perinatal psychiatry consortium (Postpartum Depression: Action Towards Causes and Treatment (PACT) Consortium 2015) have suggested PPD to have several broadly defined distinct phenotypes differentiated by the timing and severity of symptoms, complications in the perinatal period and history of mood disorders. The authors specifically suggest that the timing of symptom onset might be a useful indicator for use in future biological and genetic studies; as to date, the heterogeneity of PPD has made identification of common genes or common biomarkers difficult. Our findings are consistent with these findings and the proposed existence of a hormone-sensitive PPD phenotype (Schiller et al. 2015) suggesting that history of migraine in women with MDD may represent a group who are particularly sensitive to hormonal changes early in the postpartum.

The women in our study were recruited as part of a large on-going genetic epidemiology study of mood disorders; therefore, the sample is limited to individuals of UK White ethnicity and is likely to not be fully representative of the broader clinical population of women with MDD. Nevertheless, the strengths of this study lie in its nationwide recruitment of participants and well-characterised sample. A further potential limitation of this study is that the presence/absence of migraine was determined by self-report. The questionnaire has however previously been shown to have excellent specificity and modest sensitivity by comparing the headache diagnosis derived from the questionnaire with the clinical diagnosis of a consultant neurologist (Samaan et al. 2009). Due to sample size limitations, we were unable to stratify the migraine group further by the presence of aura or headache intensity or frequency specifically in relation to the menstrual cycle. We were also unable to examine temporal relationships between the occurrence of migraine and mood symptoms and episodes and the potential role of psychopharmacological treatments due to the cross-sectional design. Our results require replication, ideally in prospective studies with larger sample sizes which would also allow for the collection of data on current medication being used for the treatment of both migraine and MDD. Such studies would help address the clinical utility of migraine comorbidity in individualising risk of PPD in women with major depression.

## Conclusions

We have found a specific association between migraine and PPD within 6 weeks postpartum in parous women with recurrent MDD. This finding suggests women with MDD and comorbid migraine represent a group who are particularly sensitive to hormonal changes early in the postpartum. This group may be useful for future studies attempting to characterise PPD and MDD phenotypes. The refinement of such phenotypes has implications for advances in individualising treatment and future biological and genetics studies.
